# *Loa loa* Encephalopathy Following Treatment With Benzimidazole Derivatives: A Systematic Review

**DOI:** 10.1093/ofid/ofaf700

**Published:** 2025-11-24

**Authors:** Saskia Dede Davi, Tamara Nordmann, Lillian Rene Endamne, Wilfrid Ndzebe Ndoumba, Ayôla A Adegnika, Selidji T Agnandji, Bertrand Lell, Peter Gottfried Kremsner, Ghyslain Mombo-Ngoma, Rella Zoleko Manego, Michael Ramharter

**Affiliations:** Center for Tropical Medicine, Bernhard-Nocht Institute for Tropical Medicine & I. Dept. of Medicine University Medical Center Hamburg-Eppendorf, Hamburg, Germany; German Centre for Infection Research, Partner Site Hamburg-Lübeck-Borstel-Riems, Hamburg-Lübeck-Borstel-Riems, Hamburg, Germany; Center for Tropical Medicine, Bernhard-Nocht Institute for Tropical Medicine & I. Dept. of Medicine University Medical Center Hamburg-Eppendorf, Hamburg, Germany; German Centre for Infection Research, Partner Site Hamburg-Lübeck-Borstel-Riems, Hamburg-Lübeck-Borstel-Riems, Hamburg, Germany; Center for Tropical Medicine, Bernhard-Nocht Institute for Tropical Medicine & I. Dept. of Medicine University Medical Center Hamburg-Eppendorf, Hamburg, Germany; Centre de Recherches Médicales de Lambaréné, Lambaréné, Gabon Lambaréné, Gabon; Centre de Recherches Médicales de Lambaréné, Lambaréné, Gabon Lambaréné, Gabon; Institute for Tropical Medicine, University of Tübingen, Tübingen, Germany; Centre de Recherches Médicales de Lambaréné, Lambaréné, Gabon Lambaréné, Gabon; Centre de Recherches Médicales de Lambaréné, Lambaréné, Gabon Lambaréné, Gabon; Centre de Recherches Médicales de Lambaréné, Lambaréné, Gabon Lambaréné, Gabon; Institute for Tropical Medicine, University of Tübingen, Tübingen, Germany; German Center for Infection Research, Partner Site Tübingen, Tübingen, Germany; German Centre for Infection Research, Partner Site Hamburg-Lübeck-Borstel-Riems, Hamburg-Lübeck-Borstel-Riems, Hamburg, Germany; Centre de Recherches Médicales de Lambaréné, Lambaréné, Gabon Lambaréné, Gabon; Department of Implementation Research, Bernhard-Nocht Institute for Tropical Medicine, Hamburg, Germany; Center for Tropical Medicine, Bernhard-Nocht Institute for Tropical Medicine & I. Dept. of Medicine University Medical Center Hamburg-Eppendorf, Hamburg, Germany; German Centre for Infection Research, Partner Site Hamburg-Lübeck-Borstel-Riems, Hamburg-Lübeck-Borstel-Riems, Hamburg, Germany; Centre de Recherches Médicales de Lambaréné, Lambaréné, Gabon Lambaréné, Gabon; Center for Tropical Medicine, Bernhard-Nocht Institute for Tropical Medicine & I. Dept. of Medicine University Medical Center Hamburg-Eppendorf, Hamburg, Germany; German Centre for Infection Research, Partner Site Hamburg-Lübeck-Borstel-Riems, Hamburg-Lübeck-Borstel-Riems, Hamburg, Germany; Centre de Recherches Médicales de Lambaréné, Lambaréné, Gabon Lambaréné, Gabon

**Keywords:** albendazole, benzimidazole, encephalopathy, loiasis, mebendazole

## Abstract

Loiasis, a filarial vector-borne disease, is common in rural West and Central Africa. Benzimidazole derivatives albendazole and mebendazole are recommended as alternative treatments due to their perceived safety in hypermicrofilaremic patients. There is growing evidence that benzimidazoles might also lead to *Loa loa*-associated encephalopathy. In this systematic review we analyzed all available evidence of benzimidazole-associated encephalopathy.

Literature was systematically searched in PubMed, Google Scholar, and WHO-VigiBase®, including conference abstracts and consultation of experts.

Six potential cases of benzimidazole-associated encephalopathy, including 2 fatalities, were identified among microfilaremic loiasis patients. Due to the limited global use of prolonged benzimidazole regimens for loiasis, the number of encephalopathy cases identified raises significant safety concerns, challenging the rationale of their use. Further research on mechanisms and safer alternative regimens is urgently needed.

*Loa loa* is a filarial vector-borne nematode causing loiasis. The deerflies *Chrysops silacea* and *Chrysops dimidiata* transmit infectious L3 larvae of *Loa loa* during their blood meal between 10 AM and 4 PM. Loiasis is highly endemic in parts of West- and most Central African countries [1]. The pathognomonic signs of loiasis are adult worms' passage through the eye and peripheral transient angioedema known as Calabar swellings [2]. Notably, potentially life-threatening complications of loiasis have been reported, including neurological complications [3].

Loiasis can be treated with the anthelminthic drugs diethylcarbamazine, ivermectin, or benzimidazole derivatives such as albendazole or mebendazole. Diethylcarbamazine has macrofilaricidal and microfilaricidal activity against *Loa loa* and is, therefore, the drug of choice for the curative treatment of loiasis. Ivermectin is only microfilaricide [3].

Ivermectin raised attention during the 1980s and early 1990s as part of mass drug administration in controlling onchocerciasis. Cases of life-threatening encephalopathy in individuals with high microfilaremia were observed. Therefore, loiasis became the focus of public health interest, hampering the control of onchocerciasis in regions where *Loa loa* and *Onchocerca volvulus* are co-endemic [4].

Encephalopathy is a syndrome characterized by an altered consciousness that persists for more than 24 hours. It may have a variety of causes and a large spectrum of clinical signs, including lethargy and behavioral changes [5]. Encephalopathy during the natural course of infection has been reported in patients with loiasis. While the causality between spontaneous encephalopathy in chronically infected individuals is inherently challenging to ascertain, the initiation of treatment has been established as an important risk factor for loiasis-associated encephalopathy. Table 1 shows features of *Loa loa* encephalopathy reported after treatment with diethylcarbamazine or ivermectin or when occurring spontaneously. Nine cases of spontaneous encephalopathy in patients with loiasis have been reported since 1943 (Table 1) [6–8].

**Table 1.** Features of Encephalopathy in *Loa loa*-infected Patients *C-reactive Protein (CRP)

	Ivermectin-associated Encephalopathy	Diethylcarbamazine-associated Encephalopathy	Spontaneous Encephalopathy
Most important risk factor	Pretreatment microfilaremia: > 8000 mf/mL	Pretreatment microfilaremia: > 2000 mf/mL	> 30 000 mf/mL
Clinical predictors	Hemorrhages of the palpebral conjunctiva and retina, CRP* > 40 mg/l, fever >38.0°C	Hemorrhages of the palpebral conjunctiva and retina, CRP > 40 mg/l, fever >38.0°C	Hemorrhages of the palpebral conjunctiva and retina
Frequent signs and symptoms	Severe headache, confusion, lethargy, altered consciousness including coma, and urinary incontinence, motor deficit	Severe headache, confusion, lethargy, altered consciousness including coma, and urinary incontinence, motor deficit	Symptomatology is variable, violent headache, renal failure, hemiplegia or double hemiplegia, with mental disorders, functional impairment, altered consciousness including coma, potentially terminating in sudden death
Pathophysiological considerations and hypotheses	Microfilaricidal action leads to rapid killing of microfilariae, immune response to antigen release from dying parasites causes posttreatment reactions	Microfilaricidal action leads to rapid killing of microfilariae, immune response to antigen release from dying parasites causes posttreatment reactions	Intercurrent or concomitant conditions weakening the blood-brain barrier (trauma, malaria, etc), enabling microfilariae to enter the brain tissue causing inflammation and subsequent clinical symptoms.
Clinical outcomes	Ranging from full recovery to death	Ranging from full recovery to death	Ranging from full recovery to death

Treatment-associated encephalopathy was historically first reported after the administration of diethylcarbamazine, which was used on a large scale, relatively unregulated, in the first half of the 20th century [6]. Diethylcarbamazine-associated encephalopathy occurred primarily in patients with high levels (>2000 mf/mL) of *Loa loa* microfilaremia and led to cases of fatal outcomes [8]. Since the implementation of mass drug administration programs with ivermectin for the control of onchocerciasis in loiasis co-endemic regions, ivermectin is known to similarly cause *Loa loa*-associated encephalopathy. Again, pretreatment microfilaremia constitutes the major risk factor for ivermectin-associated encephalopathy. Life-threatening and fatal cases have been reported [8].

While single-dose regimens of albendazole have little to no impact on loiasis, prolonged treatment regimens of albendazole lead to a progressive, gradual decline of microfilaremia. Additionally, albendazole has shown efficacy against adult worms when administered in treatment regimens that last several weeks.

Mebendazole has limited activity in low doses or short treatment regimens. In high-dose regimens given for 21 to 45 days, mebendazole can sterilize female adult worms and kill microfilariae, leading to microfilarial clearance. Due to albendazole's higher potency and improved bioavailability, mebendazole is rarely used in treating loiasis [9].

Until today albendazole has been advocated as a safe alternative treatment option for highly microfilaremic patients due to its slow onset of action. However, case reports indicative of a temporal association between the occurrence of encephalopathy and the use of albendazole have put this concept into question. Therefore, this systematic review collates all available evidence of benzimidazole-associated encephalopathy in patients treated for loiasis to better understand the risks and benefits of benzimidazole therapy for loiasis [10, 11].

## METHODS

### Search Strategy

This systematic review was registered in PROSPERO (CRD42024597548). We performed a comprehensive literature search in PubMed and Google Scholar to identify relevant case reports and studies. The search terms included “*Loa loa* encephal*”, “albendazole induced encephal*”, “albendazole associated encephal*”, “mebendazole associated encephal*”, “mebendazole induced encephal*”, “benzimidazole induced encephal*”, “benzimidazole associated encephal*”, “*Loa loa* neurological complications” and case reports of “*Loa loa* encephalitis”, “*Loa loa* AND meningoencephal*”, “albendazole encephal* AND loiasis”, “albendazole encephal* AND *Loa loa*, “mebendazole encephal* AND *loiasis*”, “mebendazole encephal* AND *Loa loa*”. In addition to the structured literature search, references from seminal publications, conference abstracts and interviews of experts in the field were used to identify additional evidence.

To assess safety concerns of benzimidazole treatment for loiasis, we extracted data on suspected serious adverse drug reactions with albendazole and mebendazole from the WHO VigiBase® database from 1974 to 08 April 2024. VigiBase® is an international platform that contains more than 20 million anonymized cases of adverse drug reactions reported by national pharmacovigilance centers in more than 120 countries, taking part in the WHO Program for International Drug Monitoring [12]. The data extracted from the individual case safety reports included sociodemographic information (age, sex, reporter qualification, country of origin, year of report), information about the drug administration (frequency, dosage, co-medication), the reported adverse event, including seriousness and the clinical outcome. The suspected serious adverse drug reactions were classified according to the Medical Dictionary for Regulatory Activities and grouped at the System Organ Class and individual preferred term levels. We screened all available data from the WHO African Region according to the system organ class level for terms indicative of encephalopathy among those patients infected with human filariasis. These terms included altered state of consciousness, coma, local convulsions, dizziness, abnormal coordination, encephalopathy, febrile convulsion, generalized tonic-clonic seizure, head discomfort, headache, lethargy, loss of consciousness, memory impairment, mental impairment, nervous system disorder, paralysis, partial seizures, persistent postural-perceptual dizziness, seizure, slow response to stimuli, somnolence, toxic encephalopathy, abnormal behavior, agitation, confusional state, delirium, delusional disorder, disorientation, emotional disorder, hallucination (including tactile, visual, not further classified), inappropriate affect, mental disorder, mental status changes and psychotic disorder.

This approach combined a structured literature search, systematic assessment of the VigiBase®, and screening of gray literature, allowing for a thorough analysis of safety data and published literature [12].

### Inclusion Criteria

We included all case reports, case series, clinical trials, and individual safety case reports in which *Loa loa* was diagnosed and treated with benzimidazole drugs. Cases with established alternative causes for encephalopathy were excluded. Cases were classified according to the temporal association of treatment with the onset of symptoms and the evolution of typical clinical signs of treatment-associated encephalopathy as established for ivermectin and DEC (Table 1). Articles in German, English, French, and Spanish were considered.

Regarding Vigibase®, we included all individual safety case reports from the WHO Africa Region with provided data about the seriousness and treatment indication (filariasis).

### Exclusion Criteria

We excluded all studies that focused on ivermectin or diethylcarbamazine, in which benzimidazole and ivermectin or diethylcarbamazine were co-administered or did not have the target population (patients with loiasis). We further excluded all studies in which albendazole was given in a single dose or in which mebendazole was given for up to 3 days to exclude treatment regimens used on a large scale for the treatment of soil-transmitted helminths and which are known to exert no relevant effect on loiasis.

Regarding Vigibase®, we excluded all individual case safety reports outside the WHO Africa Region due to the absence of loiasis transmission outside of sub-Saharan Africa and all individual case safety reports in which other drugs than albendazole or mebendazole were administered or ivermectin and/or diethylcarbamazine were co-administered. Additionally, we excluded all individual case safety reports, for which data about the seriousness and treatment indication (filariasis or unspecified filariasis, loiasis, *Loa loa* or loaose) were unavailable. We used STATA 17.0 Basic Edition (StataCorp, Lakeway Drive, USA) analysis of data.

### Risk of Bias Assessment

We used the Joanna Briggs Institute Critical Appraisal Checklist for Case Reports (last amended in 2017) to assess the risk of bias in the chosen case reports. No other study types were included in the analysis [13].

### Data Extraction

Two investigators independently identified and screened the studies according to the inclusion and exclusion criteria. Both independently extracted data which included demographics of participants (age and sex, country of origin), treatment (drug, dose, length of treatment, number of treatment subjects), laboratory (microfilarial density (mf/mL), C-reactive Protein (CRP), eosinophilia and clinical characteristics (subjects general signs and symptoms and with neurological signs and symptoms) pretreatment and posttreatment microfilarial density, medical imaging and functional diagnostic assays, and the clinical outcome (recovery, recovery with sequelae or death).

## RESULTS

### Systematic Literature Search

**Figure 1.**
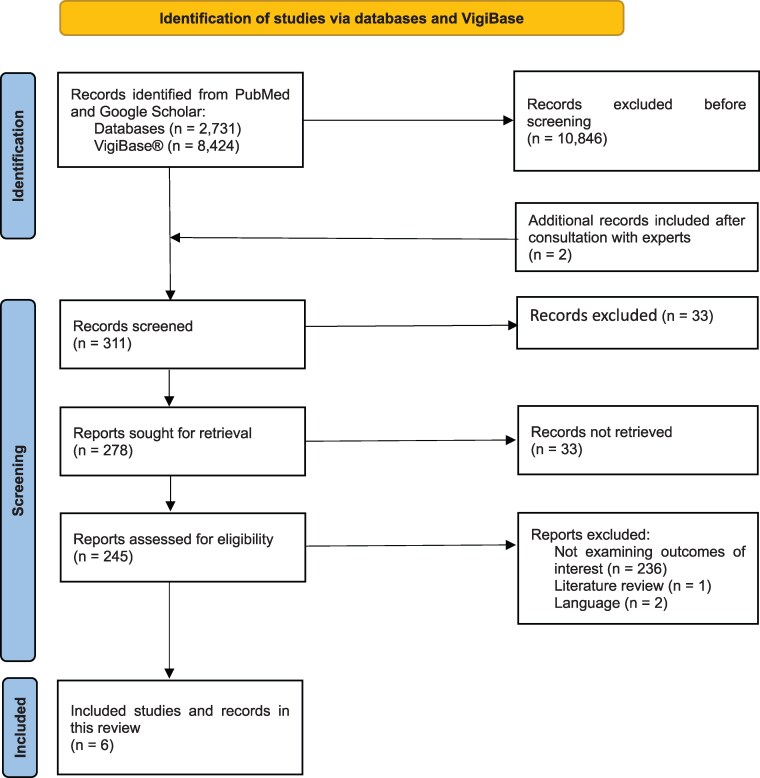
PRISMA flowchart summarising the literature search results.

Figure 1 depicts the flowchart of the identified data. Our search identified 2731 records in PubMed and Google Scholar and 8424 individual case safety reports from VigiBase®. All individual case safety reports from VigiBase® were excluded because they did not examine the outcomes of interest (n = 8419) or lacked sufficient data for appropriate assessment (n = 5).

After consultation with experts, two additional cases were included in the analysis. One case was reported as part of a randomized controlled clinical trial. The second case was an individual safety case report from a hospital register. A total of 245 studies were selected for full-text evaluation. Of these, 239 articles were excluded for the following reasons: lack of reporting of outcomes of interest (n = 236), literature reviews (n = 1), or were published in languages other than English, French, German, or Spanish (n = 2). Ultimately, we included 5 studies and 1 hospital record. All studies met the eligibility criteria and provided sufficient data, as shown in Table 2.

**Table 2.** Summary of Features Reported for Loiasis Patients Suspected to Suffer From Benzimidazole-associated Encephalopathy

	Blum et al 2001 [14]	Volpicelli et al 2020 [11]	Moliner et al 2001	Métais et al 2021 [15]	Manego et al 2023 [16]	Hospital Record
Age and sex	55 female	25 male	17 female	47 male	73 male	32 male
Country of Origin	Cameroon	Guinea-Conakry	Guinea	Cameroon	Gabon	Gabon
Pretreatment mf/mL	152 mf/mL	15 000 mf/mL	>100 000 mf/mL^a^	35 000 mf/mL	10 050 mf/mL	3800 mf/mL
Posttreatment mf/mL	Not assessed	1250 mf/mL	0	8000–10 000 mf/mL	1400 mf/mL	250 mf/mL
Inflammation markers	CRP < 5 mg/l	WBC^b^ 10 550/µL, eosinophils	Leucocytosis, eosinophilia	WBC 10 600/µL, eosinophils	CRP = 15 mg/l	CRP = 26 835 mg/l, WBC 7080/µL, eosinophils
		2870/µL		1540/µL		0,7%
Drug	Albendazole	Albendazole	Mebendazole	Albendazole	Albendazole	Albendazole
Dosage and treatment duration	2 × 200 mg/d for 21 d	2 × 200 mg/d for 21 d	Not described	400 mg/d for 9 d then discontinuation	2 × 400 mg/d for 21 d	2 × 400 mg/d for 28 d
Onset of symptoms	Day 3	Day 13	Day 30 – D31	Day 6	Day 20	Day 24
General signs and symptoms	Not described	Fatigue, headache, dizziness	Asthenia, myalgia, anorexia, fever	Asthenia, myalgia, anorexia, fever	Asthenia	Fever, asthenia, arthralgia
Onset of neurological manifestations	Day 3	Day 21	Day 30 – D31	Day 6	Day 20	Day 31
Glasgow Coma Scale (GCS)	10/15	8/15	9/15	4/15	15/15	12/15
Neurological manifestations	Speech disorder, anisocoria, non-reactive pupils, diminished reflexes	Eye movement disorders, speech disorders, hyperreflexia, positive Babinski reflex, oral automatism, distal myoclonus	Headache, meningeal signs, reduced vigilance, eye movement disorders, bilateral Babinski	Confusion, coma	Psychomotor retardation, vertigo	Psychomotor agitation, confusion, diminished reflexes, worsening of consciousness
CSF	0 mf/mL	0 mf/mL	Abundant mf/mL	60 mf/mL	Not done	Not done
Imaging modality	MRI and CT done	MRI and CT done	MRI done	MRI done	No	No
Miscellaneous	EEG done	EEG done	No	No	Alcohol abuse	Alcohol abuse
Clinical outcome	Fully recovery	Fully recovery	Partial neurological deficits	Death	Fully recovery	Death on day 36

^a^There was no pretreatment microfilaremia but a microfilaremia during treatment.

^b^White blood cell count (WBC).

### Study Characteristics

Four out of the 6 included records were case reports. The remaining 2 cases included 1 individual case safety report from a randomized controlled open-label clinical trial and 1 hospital record. The patient reported in the clinical trial has been reconsidered retrospectively as a potential albendazole-associated encephalopathy based on the above-mentioned temporal association and clinical features. All data were published between 2001 and 2021, and features of individual cases are listed in detail in Table 2.

#### Benzimidazole Treatment Associated With *Loa loa* Encephalopathy

The available case reports described several patients who developed or are suspected to have developed encephalopathy following the treatment of loiasis with benzimidazoles. Judgements by the authors have additionally been made to suggest the likelihood of developed encephalopathy following the treatment of loiasis with benzimidazoles (Table 2). Patients differed in demographic characteristics, including 2 female and 4 male patients. All patients originated from *Loa loa* endemic countries in sub-Saharan Africa and were aged between 17–73 years. Most patients had high pretreatment microfilarial load, with 4 patients having more than 8000 mf/mL. Importantly, the onset of clinical symptoms of encephalopathy was delayed by at least 20 days posttreatment initiation in 4 of 6 patients. All patients experienced similar clinical signs and symptoms, including behavioral changes, fever, speech disorder, movement disorder, altered reflexes, and general neurological impairment as evidenced by a decrease in Glasgow Coma Scale outcomes (Table 2). Worsening of consciousness, coma and death (n = 2) were the most severe outcomes of the reported benzimidazole-associated encephalopathy.

#### Risk of Bias Assessment

To address the potential for risk of bias, we applied a risk of bias assessment tool according to the Joanna Briggs Institute designed for case reports [13]. As there are no validated tools for risk of bias assessment for individual safety case reports, we applied this risk of bias assessment to the individual case safety reports. The case reports were mostly of high quality overall. One individual case safety report was moderate. Despite the application of the risk of bias, the quality of the case reports and individual case safety reports differed. All demographic and clinical characteristics, including symptoms onset and clinical outcome, were well described. However, the diagnostic approaches differed. Four out of 6 reports examined the CSF and conducted medical imaging of the brain (MRI and CT, or only MRI). Two out of 6 reported functional assessment using EEG. Most case reports had comparable laboratory parameters, such as CRP and hemograms. Pretreatment microfilaremia was missing in one report. Instead, an interim value was given. In another report, the posttreatment microfilaremia was not assessed.

## DISCUSSION

Benzimidazole-associated encephalopathy's demographic and parasitological features varied in the reported cases. Most importantly, pretreatment microfilaremia ranged between very low and very high microfilaremia (152 mf/mL— > 100 000 mf/mL) [6, 9, 10, 14–16]. Assuming the correctness of the reported parasitological results, this starkly contrasts with our current understanding of treatment-associated encephalopathy reported for diethylcarbamazine and ivermectin, where high microfilarial load constitutes the major risk factor for serious adverse reactions. Similarly, the temporal onset of signs and symptoms of encephalopathy differed considerably for benzimidazole treatment compared to encephalopathy associated with the use of diethylcarbamazine or ivermectin. Contrary to the early onset of complications within the first 48 hours after treatment with the rapidly acting diethylcarbamazine and ivermectin, complications occurred mainly between 3–4 weeks after treatment initiation with benzimidazole drugs. Importantly, albendazole therapy exerts a similar—while delayed—reduction of microfilarial load as ivermectin of ∼80%–90% at 3–4 weeks after treatment initiation. Considering the clear temporal association with benzimidazole therapy and its delayed onset of anthelminthic activity of albendazole and mebendazole against *Loa loa*, the onset of encephalopathy seems pathophysiologically plausible.

Importantly, clinical signs and symptoms of benzimidazole-associated encephalopathy were similar to reports for diethylcarbamazine and ivermectin [14]. The most frequent non-neurological symptoms include arthralgia, abdominal pain, diarrhea, fever, vomiting, diffuse hypertonia, conjunctival or retinal hemorrhage, pruritus, and renal impairment. The most common neurological symptoms were vertigo, loss of balance, speech difficulties, loss of osteotendinous reflexes, lack of response to pain, altered consciousness (such as obnubilation), and coma [6, 10, 11, 14–16].

When looking from hindsight at the 6 cases of benzimidazole-associated encephalopathy, all reports share similar features for the onset of symptoms, duration, and further temporal evolution, except for the case reported by Blum et al. This case was exceptional as the onset of clinical symptoms occurred on day 3 and, most importantly, lasted only a few hours before complete resolution. This contrasts with the other cases, which experienced the onset of symptoms later and showed a typical temporal evolution of encephalopathy over several days. We may only speculate whether the case reported by Blum et al reports benzimidazole-associated encephalopathy in the treatment of loiasis or another undiagnosed transient cognitive impairment [3, 14, 17]. Taking all these aspects into account—including the low baseline microfilaremia and the atypical time frame from treatment initiation to the onset of neurological symptoms, including the short duration of the neurological condition —we concluded that the case reported by Blum et al is unlikely to represent a benzimidazole-induced encephalopathy. Another case, which may be associated with benzimidazole treatment, but requires critical consideration, is the case reported by Métais et al [15]. In contrast to the case reported by Blum et al, almost all features are consistent with benzimidazole-associated encephalopathy, apart from the relatively early onset of symptoms on day 6 [14–16]. Typically, benzimidazole-related encephalopathy occurs 2–3 weeks after treatment initiation, which may be related to the fact that approximately 80% of the microfilarial reduction takes place within the first 4 weeks. Therefore, the case reported by Métais et al may be classified as *Loa loa*-associated encephalopathy following benzimidazole treatment. However, it may not be ruled out that given the high microfilaremia, this episode may have occurred spontaneously, or if alternative pathophysiological mechanisms beyond the direct anthelminthic activity against *Loa loa* may have contributed.

The clinical management of patients with signs of encephalopathy differed in the analyzed case reports. Treatment always included discontinuing benzimidazole therapy, but showed different approaches to supportive care. While some case reports indicate corticosteroid treatment, other patients did not receive this immunomodulatory drug [6, 10, 11, 14–16]. Intensive care was available for a subset of patients but could not prevent serious sequelae or death in all cases [6, 10, 11, 14–16]. These aspects highlight the complexity of these potentially life-threatening clinical complications.

While microfilaremia was not identified as a necessary risk factor for treatment-associated encephalopathy, most patients had a high microfilarial load. Alcohol abuse was reported for some patients and has previously been discussed as a potential risk factor for loiasis-associated encephalopathy. This aligns with analyses of loiasis encephalopathy after ivermectin treatment and may constitute an optional risk factor [18]. While alcohol intoxication remains a relevant differential diagnosis, both the hospital case and the case reported by Manego et al share key features—namely, a comparable albendazole dosage, markedly elevated pretreatment microfilaremia, and a similar day of onset of neurological symptoms—which support the plausibility of *Loa loa*-associated encephalopathy triggered by benzimidazole therapy.

This series of case reports of loiasis-encephalopathy following benzimidazole treatment raises important questions to our current understanding of the role of albendazole in the treatment armamentarium against loiasis. While to date, albendazole was considered as the only safe medical treatment option for highly microfilaremic patients to reduce microfilaremia gradually before curative treatment with diethylcarbamazine, these data seriously question this understanding and treatment approach [3]. To better appreciate the importance of this finding, the relative risk for treatment-related encephalopathy needs to be considered.

Robust estimates for the probability of developing treatment-associated encephalopathy have been established for ivermectin in patients with loiasis. The probability is closely related to the pretreatment microfilarial load. The probability of developing an ivermectin-associated encephalopathy in patients with loiasis is ≈ 2 per 1000 at a microfilarial load of 10 000 mf/mL, 30 per 1000 at a microfilarial load of 30 000 mf/mL and 620 at a microfilarial load of more than 150 000 mf/mL [19]. The situation is much less clear for benzimidazole-associated encephalopathy in patients with loiasis. While benzimidazoles are used on a massive scale to treat soil-transmitted helminths, these treatment regimens are single-dose to 3-day regimens [20]. These regimens have negligible activity against microfilaremia and the tissue-dwelling helminth *Loa loa* and, therefore, may not constitute a relevant risk factor for treatment-associated encephalopathy in loiasis patients [21, 22]. Substantial activity against *Loa loa* is only observed when benzimidazole drugs are administered over a prolonged period, which usually is between 3 and 4 weeks [3, 9, 16, 23, 24]. To our knowledge, this regimen has not been used on a large scale anywhere in the endemic world to treat loiasis patients or in control programs.

While a large but mainly unpublished patient cohort has been treated in Libreville, Gabon, to the best of our knowledge no other institutions have treated considerable numbers of patients with such prolonged benzimidazole regimens. Consequently, we must carefully evaluate the risk-benefit balance for individual treatment decisions. Patients should be informed in detail that prolonged benzimidazole-based regimens may be associated with a risk for treatment-related encephalitis and treatment decisions have to be made within a shared decision-making framework taking patient's preferences into account. Based on the published clinical trials reporting the use of benzimidazole therapy in long-term regimens, 366 (albendazole: 313, mebendazole: 53) patients have been treated with benzimidazole-containing regimens [9, 16, 21, 22, 24–28]. The number of patients treated in non-endemic regions is overall limited, and benzimidazole-based regimens are usually not the first-line treatment. Given that anthelminthic treatment is rarely prescribed for loiasis in the healthcare systems of endemic regions, we, therefore, conclude that only a very limited number of patients have so far been treated with prolonged benzimidazole-containing regimens.

Assuming based on the above-mentioned evidence that a total of 1000–10 000 patients have been treated for loiasis with prolonged regimens containing benzimidazoles and assuming 5 out of the 6 here described cases as likely being associated with benzimidazole therapy, an absolute risk for benzimidazole-associated encephalopathy of 1:200–1:2000 per patient is estimated. While the total number of patients ever treated for loiasis with prolonged benzimidazole treatment regimens remains unknown, it is clear that the substantially delayed onset of encephalopathy by several weeks following treatment with benzimidazoles inevitably leads to underreporting of cases and, thus, underestimation of the true incidence. Even if this figure would require correction by a multiplicative factor of 10—which seems highly implausible—the associated risk of treatment with benzimidazole regimens is within the magnitude of ivermectin that is unequivocally judged as contraindicated in the treatment of highly microfilaremic individuals due to its risk to induce potentially life-threatening encephalopathy [16, 29–31]. This conclusion importantly challenges our current understanding of the role of albendazole in the treatment of microfilaremic loiasis.

This systematic review has strengths and limitations that must be considered. First, the systematic and thorough search strategy, including publication databases, field experts, and WHO's safety database, ensured the identification of all published and 2 additional case reports that constitute the entire available evidence. The limited number of cases inherently limits the ability to draw definitive conclusions about potential risk factors for benzimidazole-associated encephalopathy. As most data stem from case reports and individual case safety reports, the level of evidence for epidemiological generalizability is limited. Brain imagery and CSF have not been done in 2 out of 6 cases. Thus, differential diagnoses such as neurocysticercosis cannot be completely ruled out. A potential differential diagnosis of benzimidazole-associated encephalopathy may be spontaneous *Loa loa* encephalopathy, which may occur in patients with impaired blood–cerebrospinal fluid barrier and high microfilarial densities, often exceeding 30 000/mL. Although spontaneous cases seem to be rare, they may be underestimated and may be misclassified as drug-associated encephalopathy in this review. However, this diagnosis is challenging.

In reported cases, the possibility that patients had received antifilarial therapy without disclosing it to the treating physician cannot be entirely excluded. Moreover, as loiasis is a chronic infection, it may be highly implausible that spontaneous encephalopathy would have occurred during treatment initiation. Additionally, a significant risk factor for spontaneous encephalopathy is a microfilaremia above 30 000/mL, which appears only in 2 out of the 6 reported cases. Clinically, a clear distinction between drug-associated and spontaneous encephalopathy is not possible, as both share overlapping features [6].

While the overall risk estimation needs to be taken cautiously, the temporal link between prolonged benzimidazole treatment regimens and loiasis-encephalopathy occurrence seems well substantiated.

## CONCLUSION

Benzimidazole treatment of patients with microfilaremic loiasis may be associated with life-threatening encephalopathy when used in prolonged regimens. The risk may be substantial, given the number of reported cases and the estimated number of patients treated with prolonged benzimidazole regimens. These data underscore the importance of careful clinical monitoring of loiasis patients under treatment to minimize the risk of encephalopathy. Finally, critically questioning the risk-benefit balance and indication of treating loiasis becomes necessary. The role of benzimidazoles requires to be redefined in light of these data as the understanding of their favorable safety profile in microfilaremic patients became untenable. Further research is needed to understand the underlying mechanisms of loiasis-encephalopathy and ultimately to develop safer and efficacious treatment regimens.
